# Modes of Interaction of KMT2 Histone H3 Lysine 4 Methyltransferase/COMPASS Complexes with Chromatin

**DOI:** 10.3390/cells7030017

**Published:** 2018-03-02

**Authors:** Agnieszka Bochyńska, Juliane Lüscher-Firzlaff, Bernhard Lüscher

**Affiliations:** Institute of Biochemistry and Molecular Biology, Medical School, RWTH Aachen University, Pauwelsstrasse 30, 52057 Aachen, Germany; abochynska@ukaachen.de (A.B.); jluescher-firzlaff@ukaachen.de (J.L.-F.)

**Keywords:** ASH2L, chromatin, core histone, DPY30, gene expression, histone mark, histone modification, KMT2, mixed-lineage leukemia, methylation, MLL, post-translational modification, RBBP5, transcription, WDR5

## Abstract

Regulation of gene expression is achieved by sequence-specific transcriptional regulators, which convey the information that is contained in the sequence of DNA into RNA polymerase activity. This is achieved by the recruitment of transcriptional co-factors. One of the consequences of co-factor recruitment is the control of specific properties of nucleosomes, the basic units of chromatin, and their protein components, the core histones. The main principles are to regulate the position and the characteristics of nucleosomes. The latter includes modulating the composition of core histones and their variants that are integrated into nucleosomes, and the post-translational modification of these histones referred to as histone marks. One of these marks is the methylation of lysine 4 of the core histone H3 (H3K4). While mono-methylation of H3K4 (H3K4me1) is located preferentially at active enhancers, tri-methylation (H3K4me3) is a mark found at open and potentially active promoters. Thus, H3K4 methylation is typically associated with gene transcription. The class 2 lysine methyltransferases (KMTs) are the main enzymes that methylate H3K4. KMT2 enzymes function in complexes that contain a necessary core complex composed of WDR5, RBBP5, ASH2L, and DPY30, the so-called WRAD complex. Here we discuss recent findings that try to elucidate the important question of how KMT2 complexes are recruited to specific sites on chromatin. This is embedded into short overviews of the biological functions of KMT2 complexes and the consequences of H3K4 methylation.

## 1. Introduction

Gene expression is affected by many endogenous and exogenous signals and cues that help to shape the phenotypic traits of cells and distinguish between cell types as well as different metabolic and disease states. In eukaryotes, chromatin as a dynamic and complex three-dimensional structure, controls gene expression through the precise packaging of DNA combined with many layers of signaling and regulatory factors. These complex regulations support the development of over 200 different cell types in the mammalian organism [1]. The fundamental unit of chromatin is the nucleosome, which is composed of two copies of each of the four core histones (H2A, H2B, H3, H4 and several variants thereof) and a 147 base pair fragment of genomic DNA wrapped around the histone octamer [2]. Histones are small, alkaline proteins with a globular structure, while the N-terminal tails of core histones are unstructured, highly conserved regions of these proteins. Core histones and in particular their N-terminal tails are modified by many different post-translational modifications (PTMs). A large number of histone PTMs (aka histone marks) have been described, including methylation and several others such as acetylation, acylations, phosphorylation, ADP-ribosylation, β-*N*-acetylglucosaminylation, ubiquitinylation, and sumoylation (for review [3,4]). These PTMs control the interactions of histone and their N-terminal tails with chromatin, co-factors and transcriptional regulators, and affect the stability and positioning of nucleosomes [5]. Thus, PTMs of core histones can function as road signs for transcription factors and chromatin remodeling complexes, controlling all aspects of gene transcription. Moreover, histone modifications are involved in processes such as DNA replication [6], chromatin condensation during mitosis [7], and the response to DNA damage [8]. Deregulated modification of core histones by PTMs as well as mutations in core histones that affect their modification have been linked to diseases, including various types of cancers and neurological and autoimmune disorders [9,10,11]. While for some histone marks the molecular consequences are well characterized, for many the functional relevance is only poorly defined. Moreover, the cross-talk of individual histone marks is only slowly being unraveled, leaving open many opportunities to study distinct PTMs that all together are proposed to create a histone code [12,13].

One of the best-studied PTMs of core histones is methylation of lysine (K) residues. Various lysines have been demonstrated to be mono-(me1), di-(me2), and/or tri-methylated (me3), a modification that is catalyzed by different lysine methyltransferases (KMTs), referred to as writers. Although methylation of core histones was discovered in 1964 [14], the first enzyme, SUV39H1, which was demonstrated to transfer methyl groups onto lysine 9 of histone H3 (H3K9), was identified in 2000 [15]. Finding proteins able to erase methylation marks was challenging. Initially it was speculated that methylation can only be removed when the modified core histones are recycled. However, in 2004, the first histone demethylase was discovered [16], demonstrating that lysine methylation is a reversible PTM. Since then around 30 KMTs [17] and over 20 lysine demethylases (KDMs or erasers) [18] have been discovered and their different capabilities and specificities are being characterized.

The molecular analysis of some of the histone lysine methylations has allowed categorizing their functional relevance broadly either in gene activation or in gene repression. For example, tri-methylation of H3K9 and H3K27 are typically associated with gene repression [19,20], while methylation of H3K4 and H3K36 are strongly connected with open chromatin and gene expression [21,22]. Here we will address H3K4 methylation, which is catalyzed predominantly by MLL and SET1 enzymes (summarized as KMT2 for lysine-specific methyltransferases subclass 2 [23]). These enzymes function in complexes referred to as KMT2 complexes or COMPASS (complex of proteins associated with Set1), first identified in yeast [24,25]. While yeast has one, mammals possess 6 different KMT2 complexes that have been identified. In this review, we will discuss the different mammalian KMT2 complexes. First, we will briefly summarize the functional activities of the different complexes and refer to reviews that describe various issues in more detail. In view of the importance of H3K4 methylation both at promoters and at enhancers, a relevant question is how KMT2 complexes are recruited to DNA and chromatin and how locus-specific effects are achieved. We will focus on the various mechanisms that have been described to target these complexes to specific chromosomal sites.

## 2. Methylation of Histone H3 at Lysine 4

**Figure 1:**
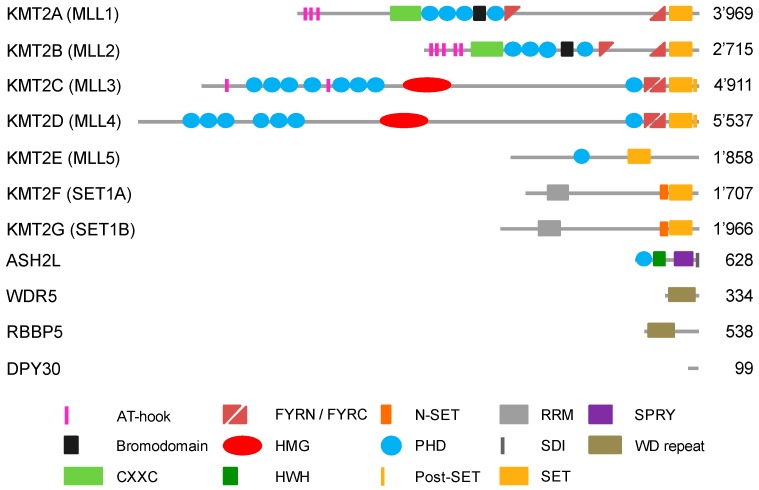
Domain organization of human KMT2 enzymes and WRAD complex proteins. AT-hooks, adenosine-thymidine-hook; CXXC, Zinc finger-CXXC domain; FYRN/C, phenylalanine and tyrosine rich region (N- and C-terminal); HMG, high mobility group; HWH, helix-wing-helix domain; N-SET, N-terminal of SET; PHD, plant homeodomain; Post-SET, C-terminal of SET; RRM RNA recognition motive; SDI, Sdc1-Dpy-30 interaction; SET, Su(var)3-9, Enhancer-of-zeste and Trithorax; SPRY, spla and the ryanodine receptor domain; WD repeat, tryptophan-aspartic acid repeat.

Lysine methylation is catalyzed by KMTs, which contain an evolutionary conserved SET domain, which was named after the first three identified proteins harboring this domain in *Drosophila*, i.e., Su(var)3-9, Enhancer-of-zeste and Trithorax [26]. SET domains transfer methyl groups from *S*-adenosyl-l-methionine (SAM) onto the N*ε* group of a lysine in substrate proteins [27]. H3K4 can be mono-, di- or tri-methylated (H3K4me1, me2 or me3, respectively) by different enzymes that include mixed-lineage leukemia (MLL1-4 or KMT2A-D, respectively) and SET1A/B (KMT2F and G, respectively) methyltransferases (MTases) (Figure 1) [25,28,29]. KMT2 enzymes are considered the main H3K4 MTases [30]. Additional enzymes described to modify H3K4 are ASH1 (absent, small, or homeotic disks protein 1), SMYD1–3 (SET and MYND domain containing 1–3), SET7/9 (SET domain containing lysine methyltransferase 7), and PRDM9 (PR/SET domain 9) (Table 1) [29]. The ASH1L protein was suggested to modify H3K4 based on peptide work [31,32]. More detailed analysis using nucleosomes as substrates and functional studies suggest strongly that ASH1L is methylating predominantly H3K36, as with H3K4 methylation a positive histone mark [33]. PRDM9 (aka Meisetz) tri-methylates H3K4 in the context of recombination [34,35]. SET7/9 can mono-methylate H3K4 on isolated histones and peptides but shows only weak activity on nucleosomes. Its main function was suggested to be in methylating non-histone proteins ([36] and references therein). SMYD1–3 methylate H3K4, however, these activities appear weak compared to other sites in core histones and non-histone substrates. Moreover, the activity towards H3K4 appears locus-specific [37,38]. Together these findings support the notion that KMT2 complexes are the main cellular H3K4 MTases controlling gene transcription (Table 1).

**Figure 2:**
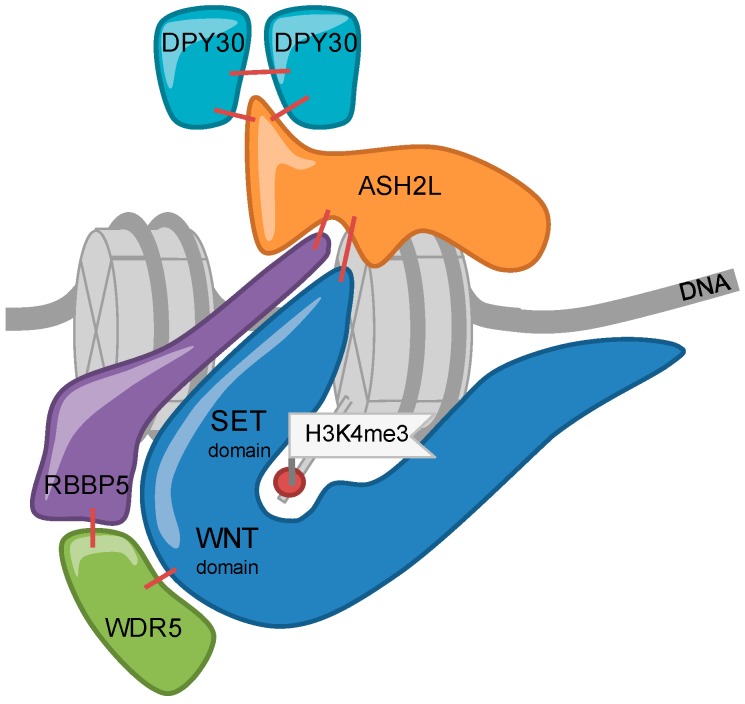
Schematic representation of the KMT2 complex. The interactions of the KMT2 enzymes with the subunits of the WRAD complex are shown by red lines [61,62,63]. For details see the text.

The catalytic subunits of KMT2 complexes show little catalytic activity when analyzed in isolation [28,30]. All 6 MTases, MLL1-4 and SET1A/B, associate in cells with the four core subunits WDR5 (WD repeat domain 5), RbBP5 (Retinoblastoma binding protein 5), ASH2L (absent, small or homeotic 2-like), and DPY30 (Dumpy-30) (or for short WRAD) (Figure 1) [50]. The WRAD complex interacts with the SET domain of KMT2 enzymes and the core subunits are required for efficient catalytic activity in vitro and in cells (Figure 2). Biochemical reconstitution experiments have demonstrated that the WRAD complex stimulates the catalytic activity of the MTases up to several hundred-fold. Thus, the WRAD complex serves as a modulatory platform [51,52,53,54,55,56,57,58,59]. In addition to regulating activity, the components of the WRAD complex are contributing to targeting KMT2 complexes to chromatin through various mechanisms (see below). Of note is also that the WRAD complex can assemble in the absence of a KMT2 subunit [52,58,60]. Interestingly, WRAD complex proteins appear to be more abundant compared to all KMT2s taken together, which supports previous findings that the WRAD complex as well as the RAD sub-complex may exist separately and carry additional functions beyond their role within KMT2 complexes [50].

Further analyses revealed that additional subunits exist that are not shared among all KMT2 complexes [50]. The unique subunits of KMT2A and B are Menin and LEDGF (lens epithelium-derived growth factor, aka PSIP1/p75) [64,65,66]. For KMT2C and D, unique subunits are PTIP (pax transactivation domain-interacting protein) and NCOA6 (nuclear receptor coactivator 6) [67,68,69]. Finally, KMT2F and G are associated with CFP1 (CxxC finger protein 1) and WDR82 (WD repeat domain 82) [50,70]. Together, these observations suggest that KMT2 complexes are divers and may elicit binding characteristics for specific regions of the chromatin and/or have additional functions beyond H3K4 methylation that presently are only poorly understood [28,71].

The above summarized unique subunits of the different KMT2 complexes suggest three subgroups, with KMT2A and B, KMT2C and D, and KMT2F and G. This is consistent with domain organization and the methylation specificity of these three subgroups (Figure 1 and Table 1). The recombinant A/B complexes are predominantly mono- and di-methylating H3K4 with low tri-methylation activity [72]. In cells, however, these complexes are important for H3K4me3 [73]. The C/D complexes are preferentially mono-methylating H3K4 [74,75,76,77,78,79]. Finally, the F/G complexes show tri-methylation activity [80]. These findings agree with the localization of KMT2 complexes in chromatin in comparison to the distribution of distinct H3K4 methylations. The F/G complexes are found at promoters as is the H3K4me3 mark [81,82]. In contrast, the C/D complexes are localized to enhancer regions, which are enriched for H3K4me1 [74,83]. Moreover, these findings are consistent with methylation of H3K4 being associated with open chromatin, i.e., as pointed out above, H3K4me1 and H3K4me2/3 as marks for accessible enhancer regions and promoters, respectively [25,84,85]. In contrast, H3K4me1 located in promoter regions is thought to have repressive functions. Whether this is due to the recruitment of KMT2 complexes that specifically mono-methylate, i.e., KMT2C and D, has not been clarified. Rather it is thought that H3K4me1 is the remaining methylation in response to the activity of demethylases that removed one or two methyl-groups from H3K4me2/3 [86].

Methylation of H3K4 is reversed by several demethylases, including LSD1 (KDM1) and four different JARID1 proteins (KDM5A-D) (Table 1) [87,88,89,90,91,92,93]. The number of enzymes capable to methylate/demethylate H3K4 supports the notion that this amino acid and its modification are important to control gene transcription.

## 3. KMT2 Complexes Are Essential for Development and Are Affected in Diseases

Knockout studies of different KMT2 family members have demonstrated the importance of these enzymes for mouse development and their functionality in different tissues. Well studied is MLL1 (KMT2A). Its homozygous deletion is embryonically lethal, whereas the *Mll1*+/− heterozygotes show retarded growth and several additional abnormalities [94,95]. Together with its well-documented role in specific forms of leukemia, it has been suggested that MLL1 is particularly relevant in the hematopoietic system [96]. Indeed, several studies have demonstrated a key role for MLL1 in hematopoietic stem and progenitor cells [97,98,99,100]. Mll2 (KMT2B), the closest homolog of Mll1, is essential for early embryonic development in the mouse [101]. However, the phenotypes are different as for example different *Hox* genes are deregulated in *Mll1* and *Mll2* knockout cells. Moreover, MLL2 is required in oocytes and during spermatogenesis but not late in embryogenesis and during homeostasis of somatic cells [42,102]. Recently, SET1A and B were also shown to be essential, again with different phenotypes [45]. This indicates that despite the homology between the different KMT2 enzymes, in particular also the high homology between SET1A and B, and between MLL1 and MLL2 (Figure 1), these MTases are functionally different. Aspects that are potentially relevant are their expression pattern, their substrate specificity also beyond H3K4, and differences in the components of the protein complexes: The latter may be particularly relevant to position KMT2 and associated factors to specific sites in the chromatin, an aspect that has been hampered at least in part by the lack of high-quality antibodies specific for the different KMT2 enzymes.

Some subunits of the WRAD complex have also been analyzed by knockout strategies in mice. *Ash2l* is essential for mouse embryonal development [103]. The embryos die very early in development at a pre-implantation stage indicating that this protein fulfills an essential function. Of note are that heterozygous animals were normal ([103] and our unpublished findings). The inducible knockout of *Ash2l* in mouse hepatocytes provokes a disintegration of the liver and subsequent death of the animals [104]. A recent study indicates that Dpy30 is necessary in hematopoiesis [105]. Loss of Dpy30 results in the accumulation of hematopoietic stem and progenitor cells, while peripheral cells are depleted. This is consistent with the analysis of Mll1, which indicated that this MTase is required for proper development of the hematopoietic system [73]. Thus, the components of the WRAD complex, which have been analyzed thus far by knockout studies, are essential for mouse and/or organ development. It is tempting to speculate that these effects are due to the activating functions of Ash2l and Dpy30 in KMT2 complexes. However, other activities might be relevant as well, including targeting to chromatin sites and functions independent of KMT2 complexes (see below).

Many of the enzymes that control H3K4 methylation are involved in diseases, most prominently cancer [28,106]. The association of KMT2 complexes with cancer is best studied with regards to MLL1 as translocations of the *MLL1* gene are associated with around 10% of human leukemias [107]. *MLL1* translocation may occur as a failure in DNA double strand repair and have been shown to provoke *MLL1* fusion to over 80 different partners. Frequently the fusion protein partners are involved, directly or indirectly, in the recruitment of DOT1L (disruptor of telomeric silencing 1-like), a H3K79 dimethyltransferase. H3K79me2 has multiple functions, both in activation and repression of gene transcription, but its role in transformation is not fully understood [108]. Some fusion partner regulate transcriptional elongation of RNA polymerase II (RNAP II) through interaction with the so-called super elongation complex (SEC) [109]. The MLL-SEC interaction stabilizes abnormally the localization of the whole complex on MLL target genes thus misregulating the transcription elongation checkpoint control (TECC) step and the release of RNAP II. The cooperation with SEC might partially explain at least some *MLL1* translocations, which have comparable phenotypes. Nevertheless, the transforming activity of some MLL fusion proteins is still elusive [110,111]. *MLL1* mutations are also linked to the rare Wiedemann-Steiner syndrome [112,113,114]. Moreover, other KMT2 methyltransferase genes have also been connected to tumor development (e.g., [115,116,117]) and mutations in *MLL2* contribute to the Kabuki syndrome (e.g., [118]). In addition to the methyltransferases, other components of the KMT2 complexes appear to contribute to cancer or cancer related processes, well documented for Menin [119,120]. ASH2L cooperates with an activated form of RAS in transforming rat embryo fibroblasts [121]. In support of a potential role of ASH2L in tumor formation, ASH2L protein but not mRNA is overexpressed in the large majority of human tumors of different origin [121,122]. Consistent with these findings, low *ASH2L* expression correlates with a favorable prognosis in patients with acute myeloid leukemia [123]. Similarly, high expression of *WDR5*, particularly when combined with high expression of *MLL1*, is correlated with high-risk acute lymphoblastic leukemia (ALL) and acute myeloid leukemia (AML) [124].

A variety of AML and ALL treatment strategies have been developed, which include the inhibition of KMT2 complexes through their interactors or fusion partners [125]. Targeting can be achieved by inhibiting the interaction with the WRAD complex, i.e., the binding to WDR5 [40,126,127], with other KMT2 complex partner including Menin [128,129] or MYC [130], as well as with DOT1L, the only known H3K79 methyltransferase [131,132]. Moreover, inhibition of the KMT2 antagonist LSD1 (lysine-specific demethylase 1) [133] and of chromatin modifiers belonging to the BET (bromodomain and extra terminal) family [134] and others [135,136] have also been suggested to be of therapeutic relevance. Through these various treatments, the proliferation of leukemic cells can be modulated, and/or the cells are sensitized to chemotherapy.

Together, the findings summarized above document the importance of KMT2 enzymes and associated factors in embryogenesis and in normal organ development and maintenance. This is further supported by the many genetic alterations associated with the genes encoding these factors that are linked to diseases.

## 4. Recruitment of KMT2 Complexes to Genes

Considering the close correlation of H3K4 methylation with open chromatin and gene expression, and of the essential functions of the KMT2 methyltransferase complexes, an important question is how specificity is achieved. In other words, how are KMT2 complexes selectively recruited to distinct sites on chromatin to catalyze gene-specific effects. Moreover, how are individual H3K4 methylation marks, i.e., mono-, di- and tri-methylation, generated at these distinct chromatin sites. H3K4me2/3 marks are positioned near transcription start site (TSS) of genes that are either actively transcribed or are in a state that allows their rapid activation [78,137]. In contrast, the distribution of H3K4me1 is broader, often at distant, active regulatory elements such as enhancers [79], while H3K4me1 appears to be a sign of gene silencing when localized in the proximity of TSSs (see also above) [86]. These locations of the different H3K4 methylation marks also relate to the question regarding their functional consequences, which we will briefly discuss below.

**Figure 3:**
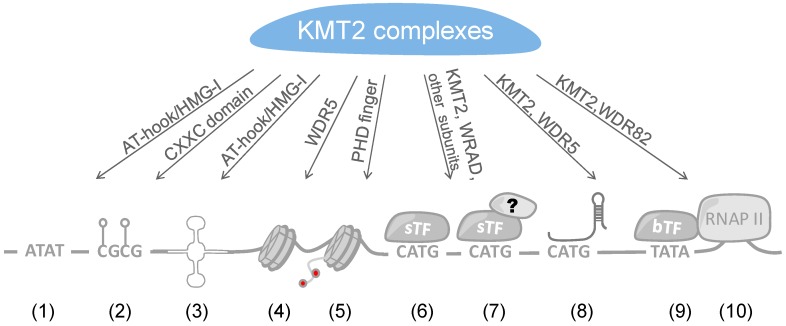
Multiple ways of recruiting KMT2s complexes to chromatin. KMT2 complex recruitment includes direct and indirect DNA interactions, possibly by more than one of these occurring cooperatively: (1) AT-rich regions recognized by AT-hooks; (2) un-methylated CpG islands by CXXC domains; (3) DNA structures by AT-hooks and HMG-I domains; (4) nucleosomes by WDR5 that interacts with the histone H3 N-terminal region; (5) nucleosomes with distinct histone marks by PHD fingers; (6) through binding to sequence specific transcription factors (sTF); (7) recruited by co-factors that bind to sTFs; (8) through long noncoding RNAs; (9) through interaction with basal transcription factors (bTF); (10) indirectly through binding to RNA polymerase II (RNAP II).

Several possibilities can be envisaged regarding how large multi-subunit KMT2 complexes are recruited to specific loci, i.e., in particular to promoters and enhancers. These possibilities include the recruitment by sequence-specific transcription factors (sTFs), specific forms of chromatin that are e.g., defined by certain patterns of histone marks, transcriptional co-factors, basal transcription factors (bTFs), and long non-coding RNAs (lncRNAs) (Figure 3). Considering the common and the unique subunits of the 6 different KMT2 complexes, site-specific recruitments are likely to occur through the latter, while the common subunits, i.e., the WRAD core complex, might contribute to stabilizing the interactions. In the following we discuss the different possibilities.

### 4.1. Recruitment of KMT2 Complexes by Sequence-Specific Transcription Factors

Arguably the most obvious mechanism is the recruitment of KMT2 complexes by sTFs, similar to many other co-factors that affect chromatin and ultimately control gene expression [138]. This would suggest that once an activating sTF has recognized its response element, e.g., as an enhancer or promoter element, binding of KMT2 complexes would represent part of the assembly of different transcriptional co-factors that modify the surrounding chromatin and contribute to the activity of a given locus (Figure 3). sTFs can bind to promoter proximal and distal response elements. It is possible that at proximal and distal sites distinct KMT2 complexes are recruited, i.e., complexes that result in H3K4me2/3 and H3K4me1, respectively. At present, little information is available whether sTFs can interact with distinct KMT2 complexes dependent on whether the factors bind near core promoters or at distal enhancers. Most studies refer to sTFs that interact at proximal sites and only few have been described to recruit KMT2 complexes to enhancer. Nevertheless, in a few cases site-specific recruitment of distinct KMT2 complexes have been noted and will be discussed below.

Considering the high complexity of the protein composition and the crowded environment in the nucleus, in which the many interactions must be controlled in a time and location-specific manner, locating multi-subunit complexes to specific sites is challenging. The use of different co-immunoprecipitation (co-IP) and chromatin immunoprecipitation (ChIP) protocols to study the interaction and distribution of factors and histone marks combined with in vitro interaction assays, and gene expression measurements are required to define causality of functionally relevant assemblies of proteins at selective sites in chromatin. Most of the studies that have reported KMT2 recruitments to chromatin do not allow to specify how the enzymes interact with DNA and chromatin, and thus some uncertainty about direct or indirect interaction remains. Also, indirect interactions might involve more than one component bridging KMT2 enzymes with DNA, an aspect that is difficult to analyze. It is worth remembering that protein-protein crosslinks with formaldehyde are less likely than protein-DNA crosslinks because of the proximity of the DNA binding domain of a sTF and the multiple sites on DNA that can be modified by formaldehyde. In contrast, stabilizing protein-protein interactions are limited by the typically limited number of sites that are in sufficient proximity for being crosslinked, which can make it difficult to detect proteins that are indirectly bound to DNA by ChIP [139].

As discussed above, the WRAD core complex appears to be associated with all KMT2 family proteins, which is necessary to assemble functional MTase complexes [50]. In that study, using label-free quantitative mass spectrometry, it was also noted that the WRAD complex components were more abundant than the KMT2 family members. Moreover, also the WRAD complex subunits appeared to have distinct cellular concentrations, suggesting that the different components are present in cells in part independent of KMT2 complexes. For example, WDR5, the most abundant WRAD component in HeLa cells, is in a more than 50-fold molar excess over all KMT2 enzymes [50]. Indeed, WRAD complex subunits associate with several additional factors indicating that these subunits participate in other protein-protein interactions and complexes [50]. Thus, these studies suggest that WRAD subunits need to be considered with care as they most certainly have additional functions, which might be relevant for the regulation of chromatin and gene expression independent of the activities of KMT2 MTases.

WDR5 interacts with multiple sTFs. During the self-renewal of embryonic stem (ES) cells, WDR5 interacts with OCT4 and possibly also with SOX2 and NANOG, suggesting that WDR5 binds to multiple transcription factors [140]. Upon differentiation of ES cells, WDR5 is substantially down-regulated correlating with a decrease in H3K4me3, suggesting that these sTFs, which are characteristic for ES cell self-renewal and pluripotency, recruit KMT2 complexes through WDR5. Of note, global H3K4me1 and 2 were increased upon WDR5 knockdown [140]. Because WDR5 is present in excess over KMT2 enzymes and other WRAD subunits [50], this may indicate a switch from preferentially tri-methylating complexes such as those containing SET1A/KMT2F and SET1B/KMT2G to other KMT2 complexes that catalyze mono- and di-methylation or possibly to other complexes without MTase activity. Moreover, OCT4 interacts through SOX2 with ASH2L, providing an additional mechanism to recruit KMT2 complexes and to regulate H3K4 methylation [141]. In ES cells, the recruitment of KMT2 complexes can also occur via SMAD2/3 TF complexes, with DPY30 playing an important role [142]. SMAD2/3 cooperates with NANOG in controlling H3K4me3. OCT4 also interacts with the C-terminal region of SET1A/KMT2F, which is necessary for H3K4 methylation [82]. This is independent of WDR5, suggesting that OCT4 has at least two modes of interacting with KMT2 complexes. These two modes might mediate separate functions. Moreover, OCT4 may bind to WDR5 or WDR5-containing complexes distinct from KMT2 complexes and thus cause unrelated activities.

The MYC/MAX TF complex also interacts with KMT2 complexes [143]. This correlates with activation of gene expression. MYC binds directly to ASH2L and to WDR5. The interaction with KMT2 complexes is mediated primarily by ASH2L, while WDR5 appears to promote DNA binding of MYC/MAX complexes to response elements independent of other WRAD subunits [143,144]. In the latter it will be interesting to determine how WDR5 interacts with DNA and what its specificity is. Because ASH2L binds to the C-terminal one-third and WDR5 to a central region of MYC, it is possible that both interactions occur simultaneously. Of note is also that both studies demonstrate that WDR5 and MYC co-localize at many promoters and that WDR5 is bound prior to MYC, indicating that WDR5 plays a role in recruiting MYC/MAX complexes to response elements, which might then affect local chromatin organization by modulating KMT2 complexes [143,144].

Mef2d is a transcription factor that is involved in murine muscle cell differentiation. Upon p38 MAPK-dependent signaling, the phosphorylation of Mef2d promotes the binding of KMT2 complexes through Ash2l [145]. This stimulates the expression of muscle-specific genes, which contribute to the differentiation of myoblasts into multi-nucleated myotubes. PRMT4 (protein arginine *N*-methyltransferase-4, aka CARM1) is an arginine-specific MTase that has been demonstrated to regulate myogenic differentiation. It was observed that PRMT4 binds to the sTFs Myogenin and Mef2c and promotes methylation of H3R17 [146,147]. A more recent finding suggests that Pax7 (paired box transcription factor) regulates *Myf5* transcription to stimulate myogenic differentiation when methylated by PRMT4. This modification of the N-terminal region of Pax7 results in binding to the C-terminal fragment of KMT2A/B (MLL1/2) [148]. This is associated with KMT2 complex recruitment and stimulation of H3K4me3 at the *Myf5* promoter.

Additional DNA binding proteins that recruit KMT2 complexes through ASH2L and control H3K4 methylation include NF-Y (nuclear transcription factor Y) [149], NF-E2 (nuclear transcription factor erythroid 2) [150,151], and USF1 (upstream transcription factor 1) [81]. Moreover, in mouse neuroblastoma cells Ash2l interacts with Ap2delta, which is expressed specifically in the developing retina and the central nervous system [152,153]. This interaction stimulates the expression of some of the canonical KMT2 target genes, including *Hoxc8* as well as genes such as *Fgfr3*, whose product is important in transcriptional regulation and signal transduction [154].

Beyond the WRAD subunits, additional proteins identified in KMT2 complexes have been described to associate with sTFs. For example, KMT2C/D (MLL3/4) interact through PTIP with Pax2 and Pax5 [50,68,69,155,156]. Similarly, the nuclear receptor coactivator NcoA6, which was found in sub-stoichiometric amounts in KMT2C/D (MLL3/4) complexes [50], targets H3K4 MTase complexes to sTFs [67,157]. These include nuclear receptors such as the farnesoid x receptor (FXR) [158] and the MAFA and B transcription factors responsible for cell-specific expression of the insulin gene in islet β-cells [159]. Similarly, the tumor suppressor and transcriptional regulator p53 may recruit KTM2 complexes through NcoA6 [160]. Other studies suggested that the histone acetyltransferase p300 is necessary for p53-KMT2 complex interaction [161,162]. Moreover, ASH2L is involved in p53-dependent gene transcription. Depletion of ASH2L interferes with target gene expression without affecting the recruitment of RNAP II, yet phosphorylation of the C-terminal domain at serine 5 was reduced, suggesting that the activation of RNAP II is dependent on ASH2L [163]. Because other subunits of WRAD are also recruited to p53 controlled promoters and H3K4me3 is enhanced, it is likely that KMT2 complexes are associating with p53. Together, these findings indicate that p53 possesses several different options to communicate with H3K4 selective MTases.

The estrogen receptor alpha (ERα) can directly interact with KMT2D/MLL4. The relevant region in KMT2D is the LXXLL motif, which is also present in KMT2C, resulting in H3K4 MTase recruitment [164]. In support of the functional relevance of this interaction, depletion of KMT2C/D but not KMT2A/B decreased estrogen-mediated induction of *HOXC6* gene expression and, complementary, depletion of ERα abolished KMT2C/D binding to the *HOXC6* promoter [165]. Yet, other observations indicate KMT2A/D or KMT2A/C as the relevant MTases in the estrogen response at *SR-B1* or *HOXB9* promoters, respectively [166,167]. Together, these analyses suggest that the interaction of ERα with different KMT2 complexes is promoter selective, possibly due to additional transcriptional regulators that determine specificity. Menin, which is only found in KMT2A/B complexes [50], can also associate with nuclear receptors such as the ER [168,169], as well as other sTFs, including c-MYB [170], thus providing an additional mechanism to recruit KMT2 complexes and control gene expression. Interestingly, amplification of the *ASH2L* locus in some breast cancers appears to result in *ASH2L* overexpression, which is positively correlated with increased expression of *ERα*. This is mediated by GATA3, which binds to an enhancer of the gene encoding ERα and recruits ASH2L and a KMT2 complex [171]. The forkhead box transcription factor FOXA1 has been described to facilitate binding of ERα to response elements in breast cancer cells [172]. Recent findings suggest that FOXA1 recruits a KMT2C complex to enhancers, controlling H3K4me1 [173]. It is postulated that this affects estrogen-dependent gene expression and cell proliferation. Together, the findings summarized here indicate that multiple interactions exist between KMT2 complexes and ERα, both direct and indirect, which control the expression, DNA binding, and function of ERα.

The E2F family of sTFs is closely associated with cell cycle-specific regulation of gene expression, for example by interacting with pocket proteins such as the tumor suppressor RB (retinoblastoma protein) [174]. Through HCF-1 (herpes simplex virus host cell factor-1) KMT2A and B, and KMT2F and G are recruited by the E2F family of transcription factors in a cell-cycle selective manner [175,176]. It has been suggested that the ANCCA/ATAD2, a AAA+ ATPase coactivator complex [177], facilitates the recruitment of KMT2 complexes [175,178,179].

Above we pointed out the transcription factors capable of interacting with KMT2 complexes, which are summarized in Table 2. Some of these factors belong to the group of pioneering TFs, including Pax7 and FOXA1. These proteins can sample genomic sites within closed chromatin. Thus, pioneering sTFs result, in the presence of additional non-pioneering factors, in the activation of promoters or enhancers that are embedded in poorly accessible chromatin. This has been well described as an important regulatory mechanism during embryogenesis and the subsequent differentiation into more specialized cell types [180]. The interaction of KMT2 complexes with pioneering factors is in support of regulatory mechanisms, in which methylation at H3K4 is important in the early stages of the activation of promoters and enhancers to allow cell type-specific chromatin opening and gene expression.

RNAP II and its interaction partners are basal transcription factors. Its positioning at the core promoter makes it an important platform to interact with transcriptional co-factors that control local chromatin as well as the chromatin along the transcribed region. WDR82 was found to interact with the C-terminal domain (CTD) of RNAP II and to mediate the recruitment of SET1A/KMT2F complexes [181]. WDR82 is one of the additional subunits, besides WRAD, that associates with some but not all KMT2s, in particular KMT2F and G complexes [50]. In yeast, but so far not analyzed in mammalian cells, the Set1 complex interacts with the CTD dependent on serine 5 phosphorylation but not when un-phosphorylated or serine 2 phosphorylated [182]. KMT2A has also been demonstrated to interact indirectly with RNAP II through hPaf1/PD2, a RNAP II-associated factor, by binding to a sequence flanking the CXXC domain of the MTase (Figure 1) [183,184]. These findings indicate that RNAP II contributes to the H3K4 methylation pattern. Indeed, it has been suggested recently that the length of binding of the Set1 complex to promoters in yeast correlates with H3K4 methylation [185]. Thus, H3K4me3 at promoters, in response to e.g., sTFs that recruit KMT2 complexes, is contributing to efficient RNAP II loading [186], but also to the efficiency of transcription in response to RNAP II-mediated methylation.

### 4.2. Recruitment of KMT2 Complexes by lncRNAs

With the advent of deep sequencing, large numbers of lncRNAs have been identified. The number of genes encoding lncRNAs appears to exceed those encoding proteins. Although typically less abundant than mRNAs, lncRNAs are frequently capped, spliced and poly-adenylated, similar to mRNAs [187,188]. lncRNAs have diverse functions including the regulation of chromatin, gene expression and signal transduction, and are associated with disease [189,190,191,192]. Interactions of lncRNAs with other macromolecules, including proteins, RNA and DNA, have been described. This offers several possibilities how lncRNAs may contribute to regulating transcription. lncRNAs can function as scaffold molecules in the organization of transcription-associated complexes and contribute to targeting of interacting proteins such as co-factors to distinct loci. This may occur by binding to sTFs, by interacting with DNA to form a triple helix or by forming R-loops [193,194,195].

Some recent reports have revealed that KMT2 complexes are linked to lncRNAs, although the information available is still sketchy (Figure 3). A first connection between KMT2 complexes and lncRNA was established when the lncRNA *HOTTIP* was identified as a regulator of the expression of the *HOXA* gene cluster [196]. The *HOTTIP* gene is located at the 5′ end of the *HOXA* cluster and interacts with the genes that are in the 5′ half of the cluster as determined by chromosome conformation capture-carbon copy (5C) analysis, indicating looping and thus close association with the promoters and regulatory sequences of these genes. The knockdown of *HOTTIP* reduces the expression of these *HOXA* genes, suggesting that the lncRNA itself is necessary for gene expression. The knockdown also correlates with reduced H3K4me3 in the 5′ half of the *HOXA* cluster. From these findings it was argued that *HOTTIP* controls histone modifications. Indeed, *HOTTIP* lncRNA interacts with the WRAD subunit WDR5 and affects H3K4 methylation [196,197]. This is consistent with a key role of KMT2 complexes in the regulation of *HOX* genes [198]. Interestingly, *HOTTIP* has been found associated with cancer (e.g., [197,199,200,201] for review [202]), possibly by affecting H3K4 methylation, which would be in support of a role of KMT2 complexes in tumors (as discussed above).

In addition to genes of the *HOXA* cluster, also *HOXB* genes are regulated by a lncRNA and KMT2 complexes. *HoxBlinc* is encoded by a gene in the *HOXB* cluster and, similar to *HOTTIP*, the knockdown or knockout of *HoxBlinc* results in reduced expression of *HOXB* genes, accompanied by a decrease in KMT2 complex recruitment and in H3K4me3 [203]. Through this mechanism, *HoxBlinc* controls hematopoietic lineage commitment, but beyond this, little is known about the biological function of this lncRNA.

*Fendrr* is another lncRNA that interacts with KMT2 complexes [204]. *Fendrr* was identified as an RNA that is essential for the development of tissues that are derived from lateral mesoderm [204]. More recent observations suggest a role of *Fendrr* in tumorigenesis [205,206,207]. Mechanistic studies indicate that the loss of *Fendrr* promotes H3K4me3 and/or represses H3K27me3 at some genes. These two histone marks are important to define bivalent chromatin, which is specified by the co-occurrence of the two marks. This is characteristic for poised promoters, which can easily switch from a repressed state to either open or closed chromatin and thus allows quick adjustment to altered gene expression needs, for example in stem and cancer cells [71]. Consistent with this is the observation that *Fendrr* interact with KMT2 complexes and with polycomb repressive complex 2 (PRC2) in cells [208]. Thus, the effects in response to a loss of *Fendrr* are complex, which is reflected in the antagonistic consequences on PRC2 and KMT2 complex recruitment. As the latter was evaluated using the core component WDR5 as a surrogate of KMT2 complexes, one word of caution is indicated. Both *HOTTIP* and *Fendrr* lncRNAs have been shown to interact with WDR5 [196,204]. As discussed above, WDR5 has been reported to bind to several other proteins beyond KMT2 complexes. In particular, WDR5 interacts with many different lncRNAs, which has been suggested to occur independently of KMT2 MTases [196,209]. Mutation of the binding site for RNA in WDR5 interferes with its cellular functions without apparent effects on KMT2 complexes [209]. Thus, additional experimentation will be necessary to further evaluate the concept.

How precisely *HOTTIP*, *HoxBlinc* and *Fendrr* are controlling the activity and the targeting of KMT2 complexes to specific loci is not fully understood. It is possible that these lncRNAs are involved in the local organization of chromatin such as looping [210,211]. By positioning the respective lncRNA, one or several KMT2 complexes could be recruited to and brought into the vicinity of the regulated promoters. Whether the positioning of the lncRNAs are due to interaction with DNA, locally synthesized RNA or protein has not been resolved [193]. The lncRNAs may have scaffolding function and through this contribute to stabilizing different transcriptional co-factors, including KMT2 complexes, at enhancers and promoters [212].

While *HOTTIP* and *Fendrr* bind to WDR5, *HoxBlinc* interacts with the SET domain of some KMT2 enzymes [203]. Yeast Set1 shares with KMT2F and G an RNA recognition motive [204]. The deletion of the Set1 RNA recognition motif (RRM) causes impaired H3K4 methylation, suggesting that RNA binding is also important for recruitment and/or stabilization of the complex on chromatin in yeast [213]. Moreover, the N-SET domain of Set1 was identified as an additional RNA binding motive, which is also present in both KMT2F and G. Thus, the interaction with RNA appears to be a conserved mechanism to position KMT2 complexes on chromatin, which is potentially relevant for the site-specific targeting of these MTases.

### 4.3. Direct DNA Binding

In the two sections above, we summarized how sTFs and lncRNAs contribute to the recruitment and positioning of KMT2 complexes. An additional mode can be the direct interaction of these complexes with DNA (Figure 3). It is of interest to note that KMT2 MTases possess DNA binding domains. These include the CXXC zinc finger domain, the AT-hook region, and the high mobility group I (HMG-I) binding motif (Figure 1). The CXXC domain is present in a number of proteins and has been found to interact with non-methylated CpG-rich DNA [214]. CpG islands function frequently as promoters and in their non-methylated state correlate with H3K4me3 and histone acetylation, marks of open, transcriptionally active genes [215]. In contrast, methylation of CpG islands results typically in repression of promoter activity and gene transcription [216]. Indeed, the CXXC domains of KMT2A and B bind to non-methylated CpG sequences [217,218,219,220]. This is also consistent with KMT2A protecting CpG clusters from becoming methylated [221]. Moreover, the binding of KMT2B complexes with CpG-rich regions promotes their H3K4me3 and this correlates with increased expression at least on some genes [220]. In addition to KMT2A and B, CFP1 is another protein that contains a CXXC domain [214]. This is worth noting because CFP1 is part of KMT2G and F complexes [50]. These two MTases do not possess any recognizable DNA binding domains and thus it is possible that they use CFP1 to directly interact with CpG-rich DNA. Therefore, these findings suggest that the CXXC zinc finger domain is sufficient for KMT2 complexes to interact with non-methylated CpG-rich DNA and thus can potentially target most human promoters. An obvious question is how selectivity is achieved, in addition to methylating CpG-rich DNA [215]. Are additional factors, for example sTFs or additional chromatin marks, required for efficient KMT2 complex recruitment? Another possibility is that once a KMT2 complex is bound its activity is controlled in a promoter-specific fashion. It will be interesting to evaluate the different options.

AT-hooks and HMG domains were originally discovered in high-mobility group proteins, which are non-histone chromatin proteins. High-mobility group proteins have multiple functions in chromatin organization, in part by competing with histone H1 and by interacting with sTFs, but also by binding to RNA components [222,223]. HMG domains come in different flavors, with AT-hooks as in KMT2A and HMG-I domains as in KMT2C and D being highly related [224]. AT-hooks and HMG-I domains are short motifs that interact with the minor groove of AT-rich DNA sequences [222,225,226]. Neither individual HMG domains nor AT-hooks are usually sufficient for selectively locating proteins to DNA, but support promoter recognition and stabilize DNA binding. The HMG domain as well as AT-hooks of KMT2s can also recognize so called cruciform DNA, four way junctions that can develop during multiple processes including replication and transcription [227]. Cruciform DNA can occur when longer than 6 bp inverted repeats in DNA sequences are extruded from the super-helical DNA. Such inverted repeats have been found in many promoter regions and near replication start sites potentially explaining the localization of KMT2 complexes to these sites [227]. Furthermore, AT-rich sequences may be important for regulation as they disfavor nucleosome formation, and thus allow easier access of KMT2 complexes as well as other TFs to DNA. Human promoters and enhancers are enriched in AT base pairs in comparison to the surrounding sequences [228]. Interestingly, a feature of some HGM domains is the induction of DNA bending and thus potentially juxtaposing nonadjacent sites thereby supporting DNA looping [229]. Together, through these DNA binding modes, KMT2 complexes are proposed to influence chromatin such that other transcription factors can bind more easily, a function that might even be independent of catalytic activity.

In addition to the catalytic subunits of KMT2 complexes, the WRAD subunit ASH2L has also been shown to directly interact with DNA. The crystal structure of an N-terminal fragment of ASH2L revealed a forkhead-like helix-wing-helix (HWH) domain that binds DNA [230,231]. Mutants in the DNA binding domain of ASH2L were analyzed in cells and were demonstrated to affect both H3K4 methylation and gene expression [230,231]. Similar effects were noted after knockdown or loss of ASH2L, correlating with reduced cell proliferation [104,121,232]. The DNA binding activity of ASH2L was also used to define consensus sequences. Both in vitro and in cells G-rich elements were identified [230,232]. The ASH2L-DNA binding affinity is rather weak [230,231], suggesting that this interaction may assist the recruitment of KMT2 complexes to DNA rather than being responsible for selective binding. A word of caution is that the analyzed mutants were not tested whether they support KMT2 complex formation and/or activity. The loss of H3K4 methylation and gene expression may be a consequence of KMT2 complex destabilization due to mutant ASH2L rather than effects on DNA binding, an aspect that needs to be resolved.

The findings summarized in this section suggest that KMT2 complexes can interact directly with DNA. This has implications for co-factor functioning. While most histone modifying enzymes require sTFs for site-selective recruitment, KMT2 complexes may bind directly to DNA, for example CpG islands, and thus affect chromatin at promoters and/or enhancers independent of sTFs. This offers an additional mode for H3K4 methylation at certain loci.

### 4.4. Interaction with Chromatin

The subunits of KMT2 complexes possess domains that suggest binding to chromatin, independent of the above summarized mechanisms that allow sequence-specific binding to certain loci (Figure 3). It is conceivable that some combinations of histone and DNA marks, including H3K4 methylation, support or antagonize the interaction with KMT2 complexes. This might be relevant for example for spreading H3K4 methylation as has been proposed for H3K27 methylation by the PRC2 and for DNA methylation [233,234,235]. H3K4 methylation is prevalent at promoters and enhancers, suggesting that these histone marks are functionally important and spreading might enhance the activity of these DNA elements. Epigenetic readers such as the plant homeodomain (PHD) finger, the double tudor domain (DTD), the MBT (malignant brain tumor) domain, the PWWP (Pro-Trp-Trp-Pro) domain, and the double chromodomain (DCD) have been described to recognize and bind to methylated H3K4 by enclosing the extended methylated side chain in a hydrophobic cage [236,237,238]. Through these different domains, it is conceivable that reading H3K4 methylation has multiple functions, including propagating a chromatin state, connecting to the basal transcription machinery and to serve as a “bookmark”.

It is interesting to note that some KMT2 MTases contain PHD fingers (Figure 1). PHD fingers are structurally conserved, represented by the canonical C4HC2C/H sequence coordinating two zinc ions. For some PHD fingers high-affinity binding was observed to certain tri-methylated lysine residues of histone H3. Therefore, KMT2s might potentially recognize the histone marks they generate via their PHD fingers and propagate H3K4 methylation further on chromatin. This could serve as part of a positive feedback loop that is independent of the underlying DNA sequence. Of the many PHD fingers only PHD3 of KMT2A exhibit H3K4me3 binding properties [236]. The other PHD fingers appear not to recognize methylated H3K4 and thus might have other functions [239], including the recognition of other PTMs [240], as protein interaction domain, and ubiquitin ligase [241].

In mixed lineage leukemia, chromosomal translocations between the gene encoding KMT2A/MLL1 and one of more than 80 partner genes have been detected. These translocations result in oncogenic fusion proteins involving the N-terminal one-third of KMT2A [242]. These fusion proteins do not contain any of the PHD fingers of KMT2A. In fact, their loss appears to be important for the transforming potential of the fusion proteins, supporting the importance of PHDs for the correct recruitment to target loci [243,244]. PHD2 of KMT2A and B have E3 ubiquitin ligase activity and can ubiquitinate the core histones H3 and H4 in vitro [241]. Whether this occurs in cells or whether other substrates are targeted and what the consequences are is still poorly understood. Furthermore, PHD3 of KMT2A was reported to interact with the RRM of cyclophilin 33 (Cyp33 or PPIE) [240]. Cyp33 competes for binding with H3K4me3, thereby promoting target gene repression [245].

The function of the KMT2C and D PHD fingers are less well explored (Figure 1). A mass spectrometry screen for methylation readers did not identify any of these PHDs as direct interactors of methylated H3K4 [246]. Instead, PHD4 and 5 of KMT2D are involved in recognizing non-methylated or asymmetrically methylated arginine 3 of histone H4 (H4R3me0 or H4R3me2a, respectively). The binding to H4R3me2a is important for KMT2D localization and activity, while symmetrically methylated H4R3 (H3R3me2s) interferes with KMT2D function [239]. Finally, it appears that KMT2F and G do not to contain domains that recognize histone modifications. However, instead, they form a complex with CFP1, which possesses a CXXC DNA binding domain as mentioned before. CFP1, conserved also in yeast (Spp1), contains additionally two PHD fingers recognizing all three states of H3K4 methylation in vitro. Depletion of CFP1 reduces H3K4me3 with no apparent effect on the other methylation states [247].

KMT2A and B possess bromodomains (Figure 1). Such domains have been described as readers of acetylated lysine residues. This histone modification neutralizes the positive charge of lysine, which affects protein binding and is associated with open chromatin and active genes [248]. However, the KMT2A bromodomain, located between PHD3 and 4, does not bind acetylated lysine residues but rather seems to modulate the activity of the PHD fingers [243]. Beyond these bromodomains no additional domains in KMT2 complex subunits have been identified that recognize acetylation marks. KMT2 can cooperate with the histone acetyl transferases (HATs) CBP/p300, which possess bromodomains [143]. Nevertheless, the inability to recognize acetylation might suggest that typically acetylation is not important for recruiting KMT2 complexes. Of note is that mutations in the p300 PHD domain affect acetylation [249]. Whether this is because of altered binding to methylated histones remains to be determined. The KMT2A fusions with CBP or p300 are transforming and promote acute myeloid leukemia [250,251,252], supporting the importance of KMT2 interaction with HATs.

WD40 domains are highly conserved structures that have been recognized to be involved in functions associated with chromatin as well as many other activities in cells [253,254]. These domains are found in WDR5 and in RBBP5. At present it is not known whether the WD40 domain in RBBP5 is involved in binding to histones. For WDR5, the WD40 domain interacts with the N-terminal end of the histone H3 tail [255]. This interaction is controlled by methylation of H3R2. While non-methylated and symmetrically di-methylated R2 (H3R2me2s) allow WDR5 binding, asymmetrically methylated R2 (H3R2me2a) prevents binding. This latter observation is consistent with H3R2me2a being selectively depleted from active promoters [256,257,258]. The pocket of WDR5 that interacts with H3R2me2s binds also to the so-called WIN motif of the SET domain of KMT2 enzymes [254]. The WIN motif and the histone H3 tail compete for binding to WDR5 [259]. H3R2me2s correlates well with H3K4me3, while H3R2me2a is depleted from H3K4me3 marked promoters [256,260,261]. Thus, it remains to be clarified what the role of WDR5 bound to H3R2me2s modified tails is in promoting H3K4 methylation. It might be that in larger KMT2 complexes structural alterations in WDR5 allow binding of both H3R2me2s tails and SET domains. WDR5 participates in other protein complexes, as discussed above, and thus may exert a function at the H3 tails independently of KMT2 complexes. An additional function of WDR5 in promoting H3K4me3 was identified by studying androgen signaling. This stimulates phosphorylation of threonine 11 of histone H3. In turn H3T11Ph is recognized by WDR5, which recruits KMT2 complexes to enhance gene expression [262]. Together these findings reveal that WDR5 is cross-talking to both methylation and phosphorylation of histone H3 tails. However, clearly, more will have to be learned about the many functions WDR5 seems to possess in controlling different aspects of gene expression.

Ubiquitination has also been known to cross-talk with H3K4 methylation. Mono-ubiquitination of several sites in the core histones H2A and H2B have been noted, with H2BK120ub1 being particularly relevant for the discussion here [263,264]. Ubiquitination of H2BK120, which is catalyzed preferentially by RNF20/hBRE1, is associated with transcribed regions, and promotes gene transcription (e.g., [265,266,267,268,269]). H2BK120ub1 stimulates the tri-methylation of H3K4 [270]. Various mechanisms are being discussed to explain the cross-talk between H2BK120ub1 and H3K4me3 [264]. In one study, ASH2L was found to mediate this cross-talk by interacting with mono-ubiquitinated nucleosomes thereby stimulating MTase activity towards H3K4 [271]. Whether ASH2L is also important to mediate recruitment of KMT2 complexes to H2BK120ub1 chromatin in cells remains to be determined. Of note is that under some conditions changes in H2BK120ub1 do not correlate with modulation of H3K4me3 [272]. Together, the findings support the notion that H2BK120ub1 can promote H3K4me3, but to define the underlying molecular mechanism will require more work.

## 5. Functional Consequences of H3K4 Methylation

H3K4 methylation correlates with open, accessible chromatin and polymerase loading. This suggests that these histone marks, mono-, di- and tri-methylation of H3K4, are read by factors that translate the information of these PTMs into polymerase recruitment and activity. Different domains, including Tudor and PHD, have been defined to bind to methylated H3K4 [238,246,273]. Recently, a novel reader domain for methylated H3K4 has been described, which is referred to as CryptoTudor [274]. While other domains are specific binders of different methylation states [238,275], CryptoTudor interacts equally well with H3K4me1 and me3. CryptoTudor in BRWD2/PHIP appears to be involved in controlling H3K27ac [274], which cooperates with H3K4me3 in forming bivalent chromatin [71].

One of the first observations with a clear functional effect was the identification of TFIID as a reader of H3K4me3 [276]. TFIID is a multi-subunit complex that contains TBP (TATA-box binding protein) and thus is a key complex to position the RNAP II complex on core promoters [277,278]. TAF3, TBP-associated factor 3, was identified to bind to H3K4me3 through its PHD finger and to promote gene transcription, consistent with the high prevalence of this modification at core promoters [276,279]. The effect was enhanced when H3K9 and H3K14 were acetylated (ac), but inhibited by H3R2me2a, suggesting histone mark cross-talk.

Another H3K4me3 interactor is the nucleosome remodeling factor NURF [280], an essential chromatin remodeling complex [281]. Mapping experiments revealed that one of the PHD fingers in BPTF/NURF301 was responsible for H3K4me3 binding [280]. The knockdown of *BPTF* and of *WDR5* were phenotypically similar in developing Xenopus embryos, supporting the notion that H3K4me3 is tightly linked to NURF-dependent chromatin remodeling. Moreover, the inhibitor of growth (ING) family of proteins, which possess tumor suppressor functions, sense histone marks [282]. The PHD fingers of ING proteins interact with H3K4me3 (e.g., [283,284,285]). They can recruit HATs as well as histone deacetylase (HDAC) complexes and affect chromatin compaction [286,287]. PHF13/SPOC1 is a chromatin associated protein with a PHD domain that controls cell proliferation and differentiation [288,289]. A recent study defines PHF13 as a reader of H3K4me2/3 and, because of its ability to also interact with RNAP II that is phosphorylated at serine 5, seems to be involved in transcriptional activation and progression. Interestingly, it can also interact with bivalent chromatin and PRC2, promoting gene repression under these conditions [290].

Recent findings suggest a role for H3K4 methylation in mitosis. It is thought that H3K4me3, which is a relatively stable histone mark, is maintained during the whole cell cycle, including mitosis when the chromatin is compacted [291,292,293]. This is consistent with the finding that KMT2 complexes appear to be associated with at least some promoters during mitosis [294], and the demonstration of low level transcription during mitosis [295]. Thus, maintaining H3K4 methylation is thought to be important to preserve the gene expression pattern from the mother cell to daughter cells and KMT2 complexes behave as mitotic “bookmarking factors” [296]. As many transcription factors are released from chromatin in mitosis, after reforming the nuclear envelope and the import of TFs, H3K4 methylation marks are thought to provide information for the appropriate localization of TFs [297,298]. H3K4me2/3 is shielded in mitosis by phosphorylation of threonine 3 and 6 of histone H3 (H3T3Ph and H3T6Ph, respectively), two sites near H3K4. H3T3Ph and H3T6Ph are thought to prevent access of demethylases and more generally of PHD finger containing proteins to H3K4me3 marks [299,300,301]. Also, H3T3Ph prevents binding of TFIID [302], which is recruited by H3K4me3 [276]. Unlike H3K4me3, the enhancer mark H3K4me1 is not shielded by H3T3Ph [302,303]. Together with other histone marks, including H3K27ac and upon dephosphorylation of the neighboring threonine residues, H3K4me2/3 will guide transcription early in G1 after exiting mitosis [293].

## 6. Conclusions

The studies summarized here suggest that H3K4 methylation is important for regulating gene transcription. H3K4me3 and H3K4me1 correlate well with open chromatin at promoters and enhancers, respectively. Indeed, numerous studies have provided evidence for this, which could not all be mentioned here. Instead, we concentrated exemplarily on some of those processes, for which direct interaction with H3K4 methylated histone H3 was demonstrated. The recent findings indicate that the targeting of the key enzymes that methylate H3K4, the KMT2 or COMPASS complexes, occurs through their recruitment by sTFs, by certain chromatin marks and possibly by lncRNAs. In addition, some evidence for direct binding to DNA has been obtained. Thus, these findings make KMT2 complexes to very broadly recruited key chromatin regulators strongly associated with transcribed genes and their enhancers. Still, relatively few sTFs have been described as recruiters. It will be interesting to see whether sTFs are the main anchors for KMT2 complexes on chromatin or whether other mechanisms, including the combinations of other histone marks, are used to position these complexes onto specific loci. Although some information is available about crosstalk with other histone modifications, certainly much more will have to be learned about the interaction of H3K4 methylation with the many other histone marks that have been identified. Thus, it will be interesting to see the results of future studies that will certainly provide a more complete understanding of the key roles KMT2 complexes and H3K4 methylation play in controlling gene transcription.

## Figures and Tables

**Table 1:** Overview of enzymes with methyltransferase activity in vitro (gray) or in cells (dark grey), and demethylase activity.

Writers Able to Methylate H3K4 (Gene ID)	Me1		Me2		Me3		Erasers Able to De-Methylate H3K4 (Gene ID) [39]	Me1	Me2	Me3
MLL1 (KMT2A) (#4297)			[40] *			[41]	LSD1(KDM1A) (#23028)			
MLL2 (KMT2B) (#9757)			[40]			[42]	LSD2 (KDM1B) (#221656)			
MLL3 (KMT2C) (#58508)	[40]			[43]			JHDM1B (KDM2B) (#84678)			
MLL4 (KMT2D) (#8085)	[40]			[44]			JARID1A (KDM5A) (#5927)			
SET1A (KMT2F) (#9739)			[40]			[45]	JARID1B (KDM5B) (#10765)			
SET1B (KMT2G) (#23067)					[40]	[45]	JARID1C (KDM5C) (#8242)			
PRDM9 (Meisetz) (#56979)					[34]	[34]	JARID1D (KDM5D) (#8284)			
SET7/9 (KMT7) (#80854)			[46,47]							
SMYD3 (KMT3E) (#64754)					[46]	[48]				
SMYD1/2 (#150572/56950)			[49]							

* If a reference is given for di- or tri-methylation, lower levels of methylation can potentially also occur by this enzyme.

**Table 2:** Summary of sequence-specific transcription factors known to recruit KMT2 complexes.

Transcription Factor	References
OCT4	[140,141]
ANCCA/ATAD2	[175,178,179]
Ap2delta	[152,153]
c-MYB	[170]
E2F	[175,176]
ERα	[164]
FOXA1	[173]
FXR	[158]
MAFA and B	[159]
Mef2d	[140]
MYC/MAX	[143,144]
NANOG	[140]
NF-Y	[149]
NF-E2	[150,151]
p53	[160]
Pax2 and Pax5	[50,68,69,155,156]
Pax7	[148,173]
PRMT4	[146,147]
SOX2	[140,141]
USF1	[81]
